# Development of Print-Speech Integration in the Brain of Beginning Readers With Varying Reading Skills

**DOI:** 10.3389/fnhum.2020.00289

**Published:** 2020-08-14

**Authors:** Fang Wang, Iliana I. Karipidis, Georgette Pleisch, Gorka Fraga-González, Silvia Brem

**Affiliations:** ^1^Department of Child and Adolescent Psychiatry and Psychotherapy, University Hospital of Psychiatry, University of Zurich, Zurich, Switzerland; ^2^Department of Psychology, The Chinese University of Hong Kong, Shatin, Hong Kong; ^3^Center for Interdisciplinary Brain Sciences Research, Department of Psychiatry and Behavioral Sciences, School of Medicine, Stanford University, Stanford, CA, United States; ^4^Neuroscience Center Zurich, University of Zurich and ETH Zürich, Zurich, Switzerland

**Keywords:** audiovisual integration, developmental trajectories, developmental dyslexia, children, functional magnetic resonance imaging (fMRI), reading acquisition, functional connectivity

## Abstract

Learning print-speech sound correspondences is a crucial step at the beginning of reading acquisition and often impaired in children with developmental dyslexia. Despite increasing insight into audiovisual language processing, it remains largely unclear how integration of print and speech develops at the neural level during initial learning in the first years of schooling. To investigate this development, 32 healthy, German-speaking children at varying risk for developmental dyslexia (17 typical readers and 15 poor readers) participated in a longitudinal study including behavioral and fMRI measurements in first (T1) and second (T2) grade. We used an implicit audiovisual (AV) non-word target detection task aimed at characterizing differential activation to congruent (AVc) and incongruent (AVi) audiovisual non-word pairs. While children’s brain activation did not differ between AVc and AVi pairs in first grade, an incongruency effect (AVi > AVc) emerged in bilateral inferior temporal and superior frontal gyri in second grade. Of note, pseudoword reading performance improvements with time were associated with the development of the congruency effect (AVc > AVi) in the left posterior superior temporal gyrus (STG) from first to second grade. Finally, functional connectivity analyses indicated divergent development and reading expertise dependent coupling from the left occipito-temporal and superior temporal cortex to regions of the default mode (precuneus) and fronto-temporal language networks. Our results suggest that audiovisual integration areas as well as their functional coupling to other language areas and areas of the default mode network show a different development in poor vs. typical readers at varying familial risk for dyslexia.

## Research Highlights

- Differential processing of congruent and incongruent audiovisual nonwords emerges in second grade and is reflected by an incongruency effect in right MTG/ITG.
- Pseudoword reading improvements with time are associated with the strength of the emerging congruency effect to audiovisual nonwords in the left STG.
- Functional coupling between the left occipito-temporal and the right SPL increases in typical readers for congruent pairs from first to second grade.
- Functional coupling between the left occipito-temporal and the left IFG/STG increases in poor readers for incongruent pairs from first to second grade.

## Introduction

Linking print (graphemes) to corresponding speech sounds (phonemes) plays a crucial role in reading and reading acquisition (Blomert, 2011). When learning how to read, most individuals are able to utilize these associations to develop sight word reading (Ehri, 2005) and become fluent readers. However, children with dyslexia struggle to attain typical levels of reading fluency and often present persistent difficulties in reading and spelling (Snowling, 1980; Shaywitz et al., 1998; Shaywitz and Shaywitz, 2008). It has been suggested that a core deficit in dyslexia may be primarily associated with impairments in learning grapheme-phoneme correspondences (Blomert and Willems, 2010; Blomert, 2011; Fraga González et al., 2017) or in applying such newly learnt correspondences while reading words (Law et al., 2018; but see Nash et al., 2016, and Clayton and Hulme, 2017). Therefore, neural mechanisms of audiovisual (AV) integration have become an important focus in neuroscientific studies of reading and dyslexia (Richlan, 2019).

AV paradigms in which the congruency of written and spoken information is manipulated have frequently been used to study the integration of visual and auditory language information in the brain (Raij et al., 2000; van Atteveldt et al., 2007; Blau et al., 2010; Kronschnabel et al., 2014; McNorgan et al., 2014; Xu et al., 2018; Ye et al., 2018). In this context, the difference of neural responses to congruent vs. incongruent print-speech pairs (congruency effect), is typically used to quantify the extent of AV integration and characterize involved networks (van Atteveldt et al., 2004; Holloway et al., 2015; Richlan, 2019). Weaker congruency differences may indicate a failure to adequately automate letter–speech sound (LS) processing skills, which has been suggested to be related to lower reading scores in children (Blau et al., 2010). Several brain regions including the superior temporal gyrus (STG), superior temporal sulcus (STS) and the auditory cortex, Heschl’s gyrus (HS) and planum temporale (PT), have been consistently identified as integration sites for letters and speech sounds (Raij et al., 2000; van Atteveldt et al., 2004; Blau et al., 2009, 2010). In addition, the supramarginal gyrus (SMG) and the angular gyrus (AG) in the parietal lobe have been shown to be involved in accessing the phonological representations for written words and letter strings in both typical readers (Church et al., 2011) and in children with reading difficulties (Vandermosten et al., 2016; Xu et al., 2018).

Several neuroimaging studies over the last 10 years have suggested alterations in AV integration processes in individuals with developmental dyslexia. Aberrant congruency effects, interpreted to reflect less efficient AV integration, were found in children and adults with dyslexia (fMRI: Blau et al., 2009, 2010; simultaneous EEG-fMRI: Kronschnabel et al., 2014; event-related potentials (ERP): Froyen et al., 2011). Using fMRI, Blau et al. (2010) revealed a congruency effect in the PT and the STS in typically reading children that was weaker in children with dyslexia. Similar effects were found in a study on adults, reporting reduced congruency effects in the superior temporal cortex for poor readers compared to fluent readers (Blau et al., 2009). This evidence is complemented by ERP studies using AV oddball paradigms (Froyen et al., 2009; Žarić et al., 2014, 2015). Those studies reported early components (e.g., mismatch negativity (MMN) latency around 150 ms) that are associated with automaticity of LS integration to be absent or diminished in 8-year-old beginning readers (Froyen et al., 2009) and 9-year-old children with dyslexia (Žarić et al., 2014). The latter study also found an association between MMN and the level of reading fluency, with reduced and shorter lasting MMN in severely dysfluent but not in dysfluent readers with dyslexia and typical readers (Žarić et al., 2014). Subsequent studies suggested an association of the late negative AV ERP (LN at around 600∼750 ms) with performance in LS training (Žarić et al., 2015) and with the N170 visual ERP in a word recognition paradigm (Fraga González et al., 2017). The LN ERP was also found to be delayed in 11-year-old children compared to adult readers (Froyen et al., 2011). Altogether, these studies suggest differences in amplitude and timing of AV integration at a neural level in individuals with dyslexia.

Next to the reduced activation in brain areas critical for AV integration, dyslexia has also been associated with atypical patterns of functional connectivity (FC) between brain regions for visual and auditory language information processing (van der Mark et al., 2011; Stevens et al., 2017). Of note, many insights into orthographic-phonological network connectivity are derived from studies that used complex phonological tasks (Sandak et al., 2004; Cao et al., 2008; Buchweitz et al., 2017; Paz-Alonso et al., 2018). For example, Paz-Alonso et al. (2018) recently compared task-related FC during AV integration between adults with dyslexia and controls by using a single-word naming task with four conditions: consistent words, inconsistent words, pseudowords, and pseudohomophones. For inconsistent words, they found stronger functional coupling between the left vOT and inferior parietal cortex for adults with dyslexia vs. controls (Paz-Alonso et al., 2018). In addition, a recent study showed reduced resting state-based coupling between the occipito-temporal (OT) cortex and the posterior cingulate cortex (PCC) of the default-mode network (DMN) in children with dyslexia compared to typical readers (Buchweitz et al., 2017). This finding was discussed as indicating a state of reduced readiness for reading in children with dyslexia as compared with typical readers (Buchweitz et al., 2017). In contrary, studies examining task-based functional coupling reported stronger (Schurz et al., 2015) functional coupling of the OT cortex (and other regions of the language network) and areas linked to the DMN, such as the precuneus, in children with dyslexia, suggesting a failure in decoupling during language tasks (but see van der Mark et al., 2011). Moreover the reduced connectivity between visual association areas and prefrontal attention areas during tasks may indicate worse integration and modulation of attentional control to visual information in impaired readers (Finn et al., 2014). To summarize, functional connectivity literature suggests broader connectivity deviances in dyslexia that extend beyond the language networks (e.g., Wolf et al., 2010; Finn et al., 2014; Fraga González et al., 2016, 2018).

Of note, most studies investigating AV integration of print and speech have focused on school children after one or several years of reading instruction or on adults when reading skills are already established (e.g., van Atteveldt et al., 2004; Froyen et al., 2009, 2011; Blau et al., 2010; Boets et al., 2013; Kronschnabel et al., 2014; Žarić et al., 2014, 2015; Holloway et al., 2015). Only recently, the initial developmental stage of integrating written and spoken information was addressed by studying preschool children (Karipidis et al., 2017, 2018) and emergent readers (Plewko et al., 2018). Specifically, Plewko et al. (2018) compared AV integration of single letters in emerging readers with and without a family history of dyslexia (i.e., FHD+ and FHD-, respectively). Differences in brain activity were detected between children with and without family risk (FHD+, FHD- respectively) in first grade, at an early stage of reading acquisition. Early stages of LS integration in FHD- were reflected in higher activation for incongruent than for congruent LS pairs in the left superior temporal cortex. The opposite pattern was detected for FHD+, with higher activation for congruent than for incongruent LS pairs in the left superior temporal cortex, which was putatively interpreted as a failure in suppressing incongruent information that could lead to impairments in automation of LS integration (Plewko et al., 2018). AV integration of grapheme-phoneme correspondences has also been examined in prereading children at risk for dyslexia by mimicking the initial step of reading acquisition through artificial-letter speech sound association training (Karipidis et al., 2017). The performance of prereaders in learning novel grapheme-phoneme correspondences and the degree of AV integration in brain regions of the language network predicted early reading fluency skills in the middle of first grade (Karipidis et al., 2018). These studies highlight the relevance of studying children at risk for dyslexia, given that estimated prevalence rates in at-risk individuals increases from 3–10% to 30–65% (Pennington and Lefly, 2001). Thus, studying at-risk populations constitutes a special interest for studying the neurobiology of reading impairments.

Despite recent insights into the development of AV integration of single characters (graphemes and phonemes), still little is known about the AV processing of letter strings and its development in emerging readers. Examining responses to pronounceable, short strings of letters (i.e., nonwords) may add important information about AV processing as they have additional word-like complexity compared to single characters but do not carry semantic information that may influence graphophonological processing. Longitudinal assessments of such basic AV processes are important to improve early characterization of the neurocognitive profiles of typical and atypical reading. The current study addresses this by examining (1) the development of AV integration of nonwords in a longitudinal design with measurements in first and second grade and (2) the functional coupling of the two major components of the reading systems, the temporo-parietal system and the ventral occipito-temporal system, in children at familial risk for developmental dyslexia. Children at familial risk for dyslexia were categorized as poor and typical readers based on their pseudoword reading performance in second grade. AV integration processes were investigated by comparing the responses to congruent and incongruent AV nonword pairs in beginning readers in the middle of first grade (T1) and one year later in the middle of second grade (T2).

## Materials and Methods

### Subjects

A total of 41 healthy, German-speaking children participated in a behavioral session and completed an implicit audiovisual target detection task in a simultaneous EEG-fMRI session in the middle of 1st grade (T1) and middle of 2nd grade (T2). The fMRI data of 9 children were excluded from analyses due to data quality issues, such as excessive motion during scanning, or incomplete longitudinal data. The remaining 32 children reported normal or corrected-to-normal vision and had an estimated IQ of ≥85. These children (*n* = 32) were grouped into typical (>25th percentile; *n* = 17, mean age 6.93 ± 0.45 years; 8 girls) and poor readers (<25th percentile; *n* = 15, mean age 6.90 ± 0.47 years; 8 girls), according to their pseudoword reading performance in second grade (T2; 1 min pseudoword reading fluency test SLRT II; Moll and Landerl, 2014). Participants either had a parent with a history of reading problems based on the Adult Reading History Questionnaire (*n* = 25; ARHQ score >0.3; Lefly and Pennington, 2000) or a sibling with reading difficulties (*n* = 5) or a developmental language delay (*n* = 2). Participants’ parents received a written and an oral description of the study information and gave written informed consent.

### Behavioral Assessment

Behavioral assessments were performed in a separate session on average 5.08 ± 5.79 days and 12.47 ± 9.19 days before the imaging sessions, for T1 and T2 respectively. All children at both time points were examined on rapid automatized naming (RAN) of objects, letters, and digits, 1-min word and pseudoword reading fluency (SLRT-II; Moll and Landerl, 2014), silent sentence reading (SLS; Mayringer and Wimmer, 2003), vowel substitution (BAKO; Stock et al., 2003), as well as letter-speech sound knowledge in upper and lower case letters. Additionally, in first grade, standardized tests were performed to assess phonological awareness, including synthesis of onset and rime, phoneme synthesis, rhyming, and phoneme categorization (Test zur Erfassung der phonologischen Bewusstheit und der Benennungsgeschwindigkeit –TEPHOBE; Mayer, 2011), and nonword repetition (Mottier Test; Wild and Fleck, 2013). In second grade children’s non-verbal IQ was assessed using the CFT1-R (Weiß and Osterland, 2013). Subject characteristics and the results of the behavioral assessments are shown in Table 1.

**TABLE 1:** Group statistics.

	Typical readers	Poor readers	Test statistic
Sex (female/male)	9/8	6/9	χ^2^(1,32) = 0.54, *P* = 0.464
Handedness (right/left/ambidextrous)	13/2/2	14/1/0	χ^2^(1,32) = 1.72, *P* = 0.190
IQ estimate – CFT 1-R	100.8 ± 11.3	101.8 ± 7.0	*t*(30) = −0.27, *P* = 0.789
Familial risk for dyslexia (ARHQ)	0.37 ± 0.11(5/6/6^#^)	0.44 ± 0.12(2/3/10^#^)	*t*(30) = −1.39, *P* = 0.101
**T1**			
Age in years	6.9 ± 0.4	6.9 ± 0.5	*t*(30) = −0.31, *P* = 0.763
Phonological awareness^1^ (TEPHOBE)	46.7 ± 7.1	44.0 ± 6.9	*t*(30) = 1.09, *P* = 0.287
Vowel substitution^2^ (BAKO^a^)	4.6 ± 2.2	5.7 ± 2.5	*t*(30) = −1.40, *P* = 0.173
Letter knowledge^2^ (upper case^b^)	23.2 ± 2.2	22.2 ± 3.2	*t*(30) = 1.02, *P* = 0.318
Letter knowledge^2^ (lower case^b^)	22.9 ± 2.7	20.7 ± 4.4	*t*(30) = 1.75, *P* = 0.090
Non-word repetition^1^ (Mottier)	29.2 ± 22.2	40.9 ± 24.8	*t*(30) = −1.41, *P* = 0.169
RAN^2^-objects^c^	0.7 ± 0.1	0.6 ± 0.1	*t*(30) = 2.11, *P* = 0.044
RAN^2^-letters^c^	1.2 ± 0.3	0.9 ± 0.3	*t*(30) = 3.43, *P* = 0.002
RAN^2^-digits^c^	1.1 ± 0.3	0.9 ± 0.3	*t*(30) = 1.81, *P* = 0.081
Silent sentence reading^2^ (SLS^d^)	8.5 ± 5.9	4.3 ± 3.7	*t*(30) = 2.40, *P* = 0.024
Word reading fluency^1^ (SLRT-II)	15.3 ± 16.0	7.0 ± 7.9	*t*(30) = 1.82, *P* = 0.080
Pseudoword reading fluency^1^ (SLRT-II)	17.9 ± 8.4	9.3 ± 7.5	*t*(30) = 3.71, *P* = 0.004
In-scanner task accuracy in %	93.9 ± 7.2	92.2 ± 12	*t*(30) = 0.48, *P* = 0.636
In-scanner reaction time in ms	633.8 ± 73.2	670.8 ± 104.0	*t*(30) = −1.18, *P* = 0.249
**T2**			
Age in years	8.4 ± 0.3	8.4 ± 0.3	*t*(30) = −0.20, *P* = 0.844
Vowel substitution^2^ (BAKO^a^)	7.0 ± 1.3	6.5 ± 1.4	*t*(30) = 0.97, *P* = 0.342
Letter knowledge^2^ (upper case^b^)	25.3 ± 1.1	24.3 ± 2.0	*t*(30) = 1.82, *P* = 0.079
Letter knowledge^2^ (lower case^b^)	25.2 ± 1.3	24.1 ± 1.7	*t*(30) = 2.06, *P* = 0.048
RAN^2^-objects^c^	0.9 ± 0.2	0.8 ± 0.2	*t*(30) = 1.78, *P* = 0.081
RAN^2^-letters^c^	1.6 ± 0.4	1.3 ± 0.3	*t*(30) = 2.56, *P* = 0.018
RAN^2^-digits^c^	1.55 ± 0.4	1.2 ± 0.4	*t*(30) = 1.76, *P* = 0.091
Silent Sentence Reading (SLS^d^)	27.65 ± 6.3	15.87 ± 5.9	*t*(30) = −2.04, *P* = 0.050
Word reading fluency^1^ (SLRT-II)	32.8 ± 16.8	15.1 ± 8.1	*t*(30) = 3.71, *P* = 0.001*
Pseudoword reading fluency^1^ (SLRT-II)	26.5 ± 7.8	15.5 ± 6.6	*t*(30) = 4.27, *P* < 0.001*
In-scanner task accuracy in %	97.51 ± 4.2	97.50 ± 2.6	*t*(30) = 0.01, *P* = 0.991
In-scanner reaction time in ms	585.2 ± 81.6	615.3 ± 95.9	*t*(30) = −0.96, *P* = 0.346

Values are mean ± standard deviation. ^1^percentile scores based on age-matched norms; ^2^raw values; ^a^number of words with correctly substituted vowels (max. = 8); ^b^number of correctly named letter-speech sounds (max. = 26); ^c^items named per second; ^d^number of sentences correctly read in 3 min (max. = 100); T1, first grade; T2, second grade; *Bonferroni corrected for 28 comparisons (P < 0.0017); ^#^number of children with different ARHQ risk scores per group: no risk (ARHQ < 0.3)/moderate risk (0.3 < ARHQ < 0.4)/ high risk (ARHQ > 0.4).

### Task

**FIGURE 1:**
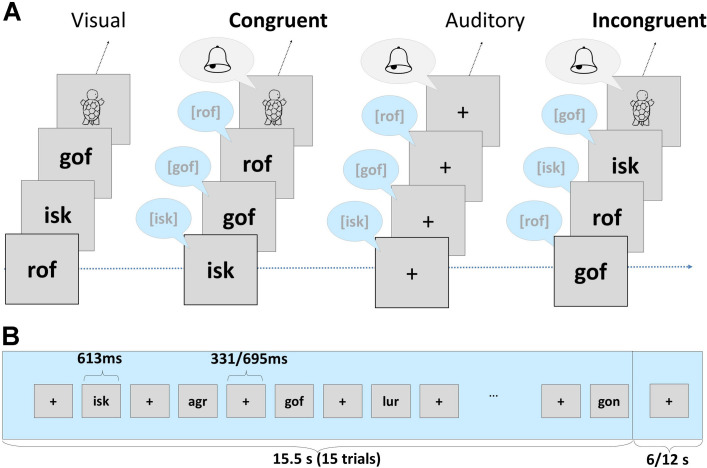
**(A)** Examples of nonwords presented in the experiment, including four conditions: unimodal visual, bimodal congruent (AVc), unimodal auditory and bimodal incongruent (AVi). Children had to press the response button whenever a visual, auditory or audiovisual target appeared (turtle, bell chime). **(B)** Illustration of the sequence and timing of one stimulation block. Each trial of the implicit audiovisual task began with the presentation of a fixation cross for 331 or 695 ms followed by a stimulus or target presentation for 613 ms. After 15 trials, a long fixation period of 6 or 12 s was presented (c.f. Karipidis et al., 2017, 2018; Pleisch et al., 2019a).

In the neuroimaging session, we employed an implicit target detection task, which was divided into four separate parts. The four parts included the presentation of letters, false font characters (Karipidis et al., 2017, 2018; Pleisch et al., 2019a), digits and short pronounceable nonwords. In this article, we focus on the processing of short pronounceable nonwords and the analyses of fMRI data. A set of 18 non-word trigrams (e.g., “rof,” “isk,” “gon”) was presented in an auditory and visual, and in a bimodal congruent and incongruent fashion using Presentation^®^ (Version 16.4)^1^, forming four different conditions: unimodal auditory, unimodal visual, bimodal congruent (AVc) and bimodal incongruent (AVi). During bimodal conditions (AVc, AVi), written and spoken nonwords were presented simultaneously. Each condition included four stimulation blocks, resulting in 16 unimodal and bimodal blocks in total. Unimodal and bimodal blocks alternated pseudorandomly and were separated by fixation periods of 6 or 12 s. Within each block, 15 items were presented randomly with an interstimulus interval of 331 or 695 ms, and each stimulus was presented for 613 ms, resulting in a block duration of 15.5 s (a schematic of the task is presented in Figure 1, for further details see Karipidis et al., 2017).

In order to maintain children’s attention, they responded by button press to auditory, visual, and audiovisual targets (six targets/condition). A drawing of a turtle and the sound of a bell chime presented either unimodally or bimodally were used as targets (Figure 1A). In total, each condition included 54 stimuli and 6 targets.

The visual information was presented in black on a gray background using video goggles (VisuaStimDigital, Resonance Technology, Northride, CA, United States), nonwords spoken by a female speaker were digitally recorded (sampling rate: 44.1 kHz; 32 bit) and normalized in Audacity (±1 dB). To ensure high quality of the auditory stimulation we used in-ear headphones (MR confon GmbH, Magdeburg) for sound presentation and the acoustic noise of the MRI was reduced by sound-absorbing over-ear headphones, a sound-absorbing mat placed in the MRI bore and a SofTone MR-sequence. Furthermore, sound volume was adjusted individually and a custom-made head pad for the EEG net was used to ensure comfort and to reduce head movement.

### Behavioral Analysis

For each behavioral assessment, differences between typical and poor reading groups were tested using independent sample *t*-tests. Behavioral responses during the neuroimaging task served to monitor children’s attention. Separate repeated measure ANOVAs were computed for reaction time and accuracy in the AV target detection task, with the within subject factor time (T1 vs. T2) and between subject factor group (typical vs. poor; Table 1). A statistical threshold of *P* < 0.05 was used, providing a critical threshold of *P* < 0.0017 after Bonferroni correction for a total of 28 *t*-test comparisons. Significant *P*-values below the Bonferroni corrected threshold are marked with an asterisk in Table 1.

### fMRI Data Acquisition

Functional MRI data were acquired using a T2^∗^-weighted whole-brain gradient-echo planar image sequence on a Philips Achieva 3T scanner (Best, Netherlands) using a 32-channel head coil with the following parameters: slices/volume: 31, repetition time TR: 1.98 s, echo time TE: 30 ms, slice thickness: 3.5 mm, slice gap: 0.5 mm, flip angle: 80^*o*^, field of view: 240 mm^2^ × 240 mm^2^, in-plane resolution: 3 mm^2^ × 3 mm^2^ × 3 mm^2^, SofTone factor: 3, sensitivity-encoding (SENSE) reduction factor: 2.2. In addition, a field map was recorded to perform B0 field map correction. To improve normalization, T1-weighted images were recorded with the following scan parameters: TR = 6.8 s, TE = 3.2 s, voxel size = 1 mm^3^ × 1 mm^3^ × 1 mm^3^, flip angle = 9°, FOV: 27 cm^2^ × 25.4 cm^2^, number of slices = 176.

### fMRI Data Preprocessing

Image processing was carried out using SPM12 on MATLAB R2016b. Preprocessing steps included B0 field map correction, realignment and unwarping, slice-time correction, coregistration and segmentation. The deformations derived from the segmentation and a pediatric brain template (age range 5.9–8.5 years) created using the Template-O-Matic toolbox (Wilke et al., 2008) were used for normalization. Voxels were resampled to 3 × 3 × 3 mm^3^ and smoothed with a full-width-half-maximum Gaussian kernel of 8 mm. Volumes with scan-to-scan motion over 1.5 mm/TR were repaired using linear interpolation between the nearest unrepaired scans using the ArtRepair toolbox (Mazaika et al., 2007). In case of more than two consecutive scans affected by movement, motion scrubbing was performed by modeling the affected volumes in an additional regressor of no interest. Data sets with more than 10% of the scans exceeding a scan-to-scan motion threshold of 1.5 mm/TR were excluded from further analyses. For a detailed description of mean and SD scan to scan head motion at each time point and for each group we refer to Supplementary Table S1.

### fMRI Whole Brain Statistical Analyses

A random-effect generalized linear model (GLM) was calculated with six predictors (auditory, visual, AV congruent, AV incongruent, target, and response) and six movement parameters for each participant, separately for first and second grade. When motion scrubbing was performed, an additional regressor of no interest was introduced to the GLM. First-level analyses on subject level included the contrasts of each condition against baseline and the comparisons between the four conditions (i.e., AV congruent, AV incongruent, visual, auditory).

In order to investigate the development of AV integration and how it differs between children with typical and poor reading skills, the analysis focused on the two audiovisual conditions, AV congruent (AVc) and AV incongruent (AVi). For each time point and using subject-wise contrast maps of AVc and AVi, we computed a 2 × 2 repeated-measure ANOVA with the within subject factor congruency (AVc vs. AVi) and between subject factor group (typical vs. poor). In addition, contrast maps of [AVc vs. AVi] were used to calculate a 2 × 2 ANOVA with within subject factor time (T1 vs. T2) and between subject factor group (typical vs. poor).

In order to test the association between AV integration and developmental changes in reading performance, multiple regression analyses of the contrast [AVc - Avi] and the pseudoword reading performance improvement from first to second grade (ΔT = T2 - T1) were computed separately for first and second grade. To examine whether the development of audiovisual integration was related to changes in reading performance, multiple regression analysis of the difference of [AVc - AVi in T2] vs. [AVc - AVi in T1] and the reading performance improvement from first to second grade was performed. Reading fluency in the 1-min pseudoword reading test was used to quantify reading performance. This allowed to investigate the association between decoding skills during reading and AV integration of nonwords. For all whole brain analyses we applied a voxel-wise uncorrected threshold of *P* < 0.005, with cluster extent threshold of 50 voxels similar to other studies involving pediatric subjects (Wimmer et al., 2010; Raschle et al., 2012; Dȩbska et al., 2016; Wang et al., 2018).

### Functional Connectivity Analyses

Functional connectivity (FC) analysis was performed with seed-voxel correlation mapping using the CONN toolbox, which is an open-source Matlab/SPM-based software for the computation, display, and analysis of functional connectivity MRI (Whitfield-Gabrieli and Nieto-Castanon, 2012). Each participant’s T1 normalized anatomical image was segmented into white matter (WM), gray matter (GM) and cerebrospinal fluid (CSF) masks. Preprocessed functional data were imported and a band-pass filter of 0.008–0.09 Hz was applied. WM, CSF, and realignment parameters were entered as confounds and regressed out from the BOLD time series, following the CompCor strategy (Behzadi et al., 2007) as implemented in CONN. Seed regions were defined in the left vOT and the left STS given the involvement of these regions in reading and AV processing of language in previous studies. Literature-based spherical seed regions with a radius of 8 mm were created using MarsBaR (Brett et al., 2002) in the vOT, MNI coordinates *x* = −44 *y* = −57 *z* = −15, (Vandermosten et al., 2016) and the left STS, MNI coordinates *x* = −60 *y* = −34 *z* = 0, (Blau et al., 2010). Mean time series were obtained by averaging the time series of all voxels in the seed region, and Pearson correlation coefficients were calculated between the mean time series of the seed and the time series of every other voxel in the brain.

Correlation coefficients were Fisher z transformed on the first-level and submitted to the second-level repeated-measure ANOVA to identify the brain regions that showed significant changes in functional connectivity of the congruency effect with the seed regions. Contrast maps of [AVc vs. AVi] were used to compute second-level ANOVAs with factors time (T1 vs. T2) and group (typical vs. poor readers) for each seed region. Subsequently, we performed additional ANOVAs for each seed region to investigate (1) the developmental trajectories from first to second grade of how functional connectivity of audiovisual integration (i.e., congruency contrast: AVc vs. AVi) changes in poor and typical readers, and (2) the difference of functional connectivity of audiovisual integration (AVc vs. AVi) between typical and poor readers in first and second grade, respectively. This approach allowed for specifically comparing connectivity patterns of different time points as well as different groups during audiovisual integration (AVc vs. AVi). Significant results are reported at a cluster extent family-wise error (FWE)-corrected *P*_*FWEc*_ < 0.05 on a cluster defining threshold (CDT) of *P_*unc*_* < 0.001.

## Results

### Behavioral Results

Group comparisons in first (T1) and second (T2) grade by definition showed significantly higher scores for typical readers as compared to poor readers for pseudoword reading fluency in T2 (*P* < 0.001; Table 1). The same group difference was also found for word reading fluency in T2 (*P* = 0.001; Table 1). The two groups did not significantly differ on the non-verbal IQ estimate (*P* = 0.789) or any other measure.

For the fMRI task, the two repeated measure ANOVAs revealed faster in-scanner reaction time (RT) [*F*(1,30) = 16.923, *P* < 0.001] and higher accuracy (ACC) [*F*(1,30) = 6.687, *P* = 0.015] for second graders than first graders. Neither the main effect of group [RT: *F*(1,30) = 1.357, *P* = 0.253; ACC: *F*(1,30) = 0.184, *P* = 0.671] nor the interaction effect of time and group were significant [RT: *F*(1,30) = 0.076, *P* = 0.785; ACC: *F*(1,30) = 0.225, *P* = 0.639].

### fMRI Results – Whole Brain Analyses

**FIGURE 2:**
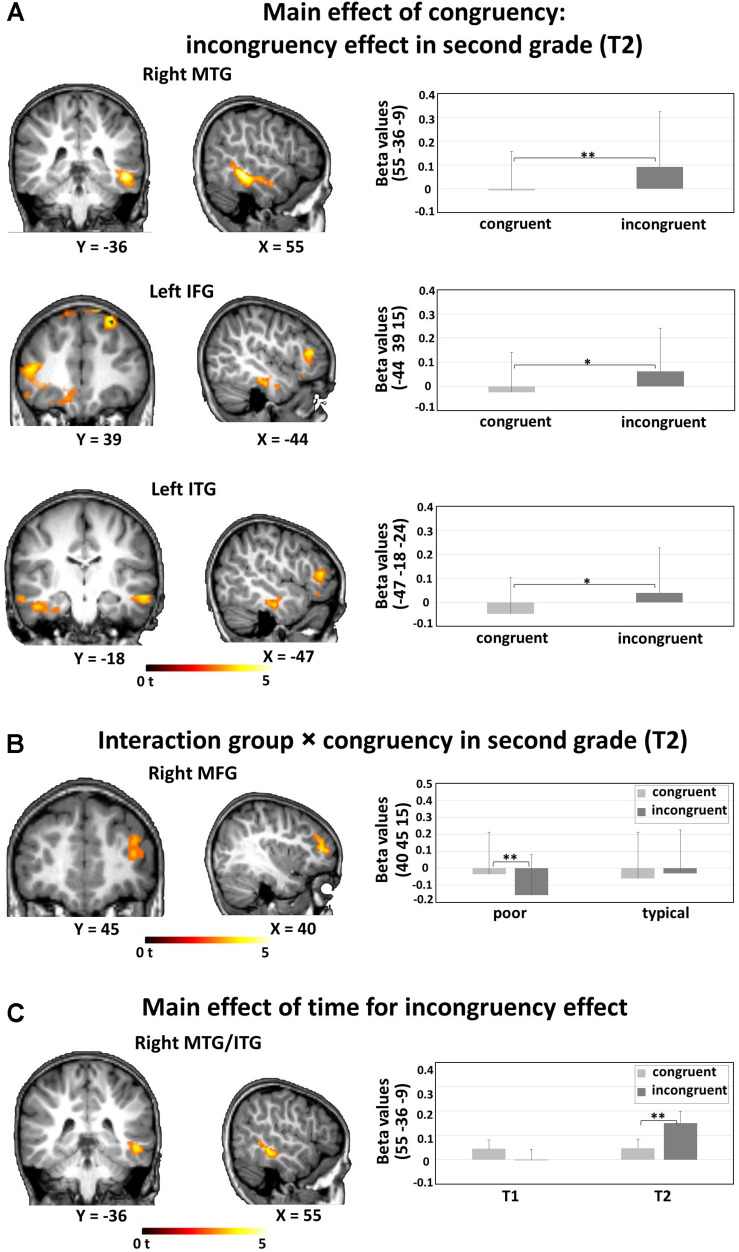
fMRI whole brain analyses: **(A)** Main effect of congruency: incongruency effect in second grade (T2). Stronger activations for incongruent (AVi) than congruent (AVc) audiovisual pairs and thus incongruency effects were found in the right MTG, the left IFG, and the left ITG. **(B)** Interaction group × congruency in second grade (T2). Significantly decreased activation was found for AVi compared with AVc in the right MFG in poor but not in typical readers. **(C)** Main effect of time for incongruency effect. An incongruency effect [AVi vs. AVc] was found in the right MTG/ITG in T2 but not in T1. A voxel-wise uncorrected threshold of *P* < 0.005, *k* > 50 was applied. Bar plots illustrate post hoc *t*-tests, asterisks denote *P*-values (*P < 0.05, **P < 0.01), and error bars illustrate standard deviations. MTG, middle temporal gyrus; IFG, inferior frontal gyrus; ITG, inferior temporal gyrus; MFG, middle frontal gyrus.

The 2 × 2 voxel-wise ANOVA on whole-brain level, with within subject factor congruency (AVc vs. AVi) and between subject factor reading group (typical vs. poor) at T1 showed no significant main effects or interactions. The same ANOVA for T2 resulted in a significant main effect of congruency and an interaction of congruency and group. Congruent (AVc) presentations resulted in weaker BOLD responses than incongruent (AVi) ones (i.e., incongruency effect) bilaterally in the middle/inferior temporal gyri, including parts of the fusiform gyrus, bilaterally in the superior frontal gyrus, in the left inferior frontal gyrus, and in the right medial frontal gyrus (Table 2A and Figure 2A). The interaction effect of congruency by group was significant in the right middle frontal gyrus: poor reading children showed a significant difference in the form of a more negative BOLD signal for AVi than AVc pairs (Table 2B and Figure 2B), while no significant difference was detected between the negative BOLD signals for AVi and AVc pairs in typical readers. When examining the incongruency effect (AVi vs. AVc) across time, the ANOVA with factors group and time yielded a significant main effect of time in the right middle/inferior temporal gyrus, showing a stronger incongruency effect in T2 than in T1. Post hoc t-tests revealed stronger activations for AVi than AVc in T2 but no significant difference for the opposite pattern, i.e., stronger activations for AVc vs. AVi in T1 (Table 2C and Figure 2C).

**TABLE 2:** Statistics of whole brain fMRI analyses.

Brain area	Hemisphere	MNI coordinates			Voxels	*T*-value	*P*-value
		*x*	*y*	*z*			
**A. Main effect of congruency in T2: incongruency effect (AVi vs. AVc)**							
MTG/ITG	Right	55	−36	−9	280	5.04	0.010*
SFG	Right	25	36	57	246	4.25	0.019*
IFG	Left	−44	39	15	220	4.25	0.032*
ITG	Left	−47	−18	−24	102	3.77	<0.001
SFG	Left	−20	21	60	88	3.57	<0.001
Medial frontal gyrus	Right	13	54	12	85	4.05	<0.001
undefined	Left	−65	9	−12	84	4.35	<0.001
**B. Interaction group x congruency in T2**							
MFG	Right	40	45	15	113	3.73	<0.001
**C. Main effect of time for incongruency effect (AVi vs. AVc)**							
MTG/ITG	Right	55	−36	−9	197	4.69	<0.001
**D. Multiple regression: congruency effect at T1 and pseudoword reading (T2-T1) performance improvement**							
SPL	Left	−26	−57	33	143	3.82	0.001
SPL	Right	28	−69	48	56	3.52	0.001
MOG	Left	−16	−105	3	52	3.53	0.001
**E. Multiple regression: congruency effect at T2 and pseudoword reading (T2-T1) performance improvement**							
STG	Right	37	−54	21	160	3.75	<0.001
PostCG	Left	−14	−42	72	95	3.74	<0.001
MFG	Left	−32	42	15	85	3.74	<0.001
STG	Left	−62	−48	15	80	4.16	<0.001
MOG	Right	37	−69	6	60	4.04	<0.001
**F. Multiple regression: congruency effect difference (T2-T1) and pseudoword reading (T2-T1) performance improvement**							
STG	Left	−59	−57	18	78	4.31	<0.001

Listed are the local maxima of significant clusters at a voxel-wise uncorrected threshold of P < 0.005, k > 50. *Cluster-level FWE corrected P-value.

**FIGURE 3:**
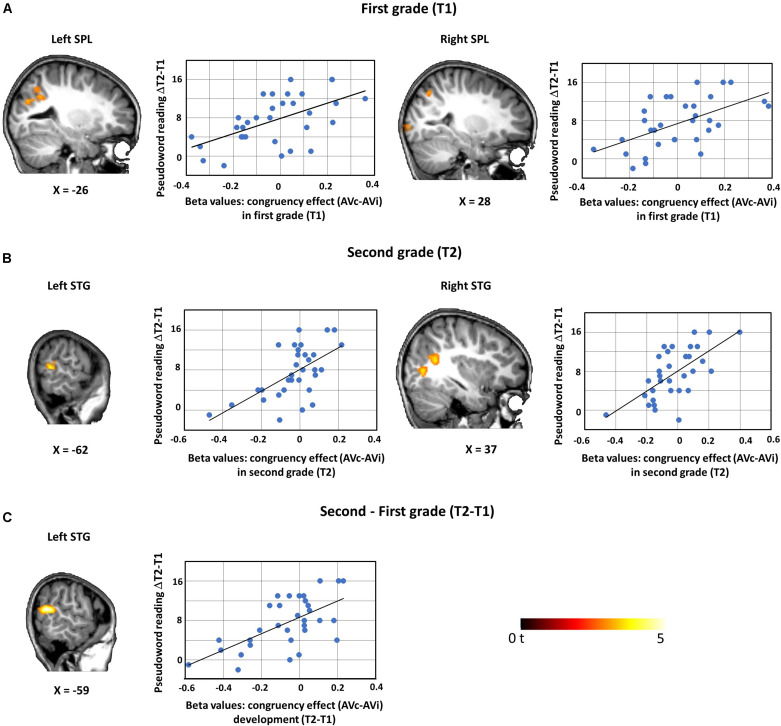
Whole-brain multiple regression analyses with pseudoword reading improvement (ΔT = T2-T1). Beta values of regions showing a significant association are plotted against ΔT pseudoword reading scores including regression lines. Pseudoword reading performance improvement ΔT was positively associated with the BOLD signal of the congruency effect **(A)** at T1 bilaterally in the SPL (left SPL, peak MNI coordinates: *x* = −26, *y* = −57, *z* = 33; right SPL, peak MNI coordinates: *x* = 28, *y* = −69, *z* = 48), **(B)** at T2 bilaterally in the STG (left STG, peak MNI coordinates: *x* = −62, *y* = −48, *z* = 15; right STG, peak MNI coordinates: *x* = 37, *y* = −54, *z* = 21). **(C)** Pseudoword reading performance improvement (ΔT) was also positively associated with the congruency effect difference over time (T2 vs. T1) in the left STG (peak MNI coordinates: *x* = −59, *y* = −57, *z* = 18). A voxel-wise uncorrected threshold of *P* < 0.005, *k* > 50 was applied. SPL, superior parietal lobule; STG, superior temporal gyrus.

Whole-brain multiple regression analysis with the pseudoword reading performance improvement from first to second grade (pseudoword reading raw score ΔT = T2 - T1) and the congruency contrast in first grade (AVc vs. AVi in T1) yielded two significant clusters in bilateral superior parietal lobule (SPL), including parts of the angular gyri, and a significant effect in the left middle occipital gyrus (Table 2D and Figure 3A). Multiple regression analysis with the pseudoword reading fluency improvement (ΔT = T2 - T1) and the congruency effect in second grade (AVc vs. AVi in T2) yielded significant clusters bilaterally in the posterior superior temporal gyrus (STG), left postcentral gyrus, left middle frontal gyrus, and right middle occipital gyrus (Table 2E and Figure 3B). When examining the association of the congruency effect difference from first grade to second grade [(AVc vs. AVi in T2) vs. (AVc vs. AVi in T1)] with pseudoword reading performance improvement (ΔT = T2 - T1), a positive association was found in the left posterior superior temporal gyrus (STG) (Table 2F and Figure 3C). No other results exceeded the threshold of *P* < 0.005, *k* > 50.

### fMRI Results – Functional Connectivity Analyses

#### Reading-Dependent Developmental Trajectories of FC During Audiovisual Integration

**FIGURE 4:**
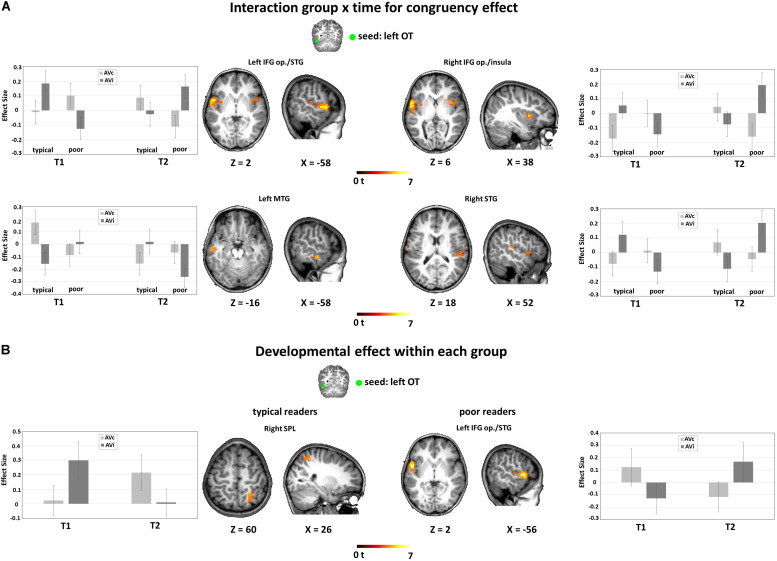
FC-fMRI analysis: Interaction between group and time for the congruency effect (AVc vs. AVi) and developmental effect within each group. **(A)** Interaction group × time for congruency effect. Significant interaction of group × time for AVc vs. AVi from the left OT seed bilaterally to the IFG op./STG (peak MNI coordinates: *x* = −58, *y* = 0, *z* = 2; *x* = 38, *y* = 6, *z* = 6; *x* = 52, *y* = −30, *z* = 18) and to the left MTG (peak MNI coordinates: *x* = −58, *y* = −16, *z* = −16). **(B)** Developmental effect within each group: main effect of time for the congruency effect in typical readers (left) and in poor readers (right). For typical readers, we found a developmental effect of FC from the left OT seed to the right SPL (peak MNI coordinates: *x* = 26, *y* = −52, *z* = 60), reflected in an increase FC for AVc pairs and a decrease of FC for AVi pairs. For poor readers, the reverse pattern was found for FC from the left OT seed to the left IFG op./STG (peak MNI coordinates: *x* = −56, *y* = 2, *z* = 2), with a developmental decrease of FC for AVc pairs and an increase for AVi pairs. Significant results are reported at a voxel-level uncorrected *P* < 0.001 and a cluster extent family-wise error (FWE)-corrected *P_*FWEc*_* < 0.05. FC, functional connectivity, OT, occipito-temporal, SPL, superior parietal lobule, STG, superior temporal gyrus, MTG, middle temporal gyrus, IFG op, inferior frontal gyrus pars opercularis.

For the left OT seed, the ANOVA using the congruency contrast (AVc vs. AVi) revealed a significant interaction of group by time in large bilateral clusters around the rolandic operculum spanning the IFG pars opercularis (IFG op.), the superior temporal gyrus and the insula, and in further clusters located in the left MTG, and in the caudal part of the right anterior cingulate (Figure 4A and Table 3A). For the left STS seed, we found a significant main effect of group for connectivity to the bilateral STG, as well as to the right postcentral gyrus and posterior cingulate (Table 3B).

**TABLE 3:** Statistics of FC-fMRI analyses.

Brain area	Hemisphere	MNI coordinates			Voxels	*T*-value	*P*-value
		*x*	*y*	*z*			
**A. Interaction group × time**							
**Seed: left OT**							
IFG op./ STG	Left	−58	0	2	834	6.02	<0.001
IFG op/Insula	Right	38	6	6	311	5.41	<0.001
STG	Right	52	−30	18	183	4.33	0.008
MTG	Left	−58	−16	−16	161	4.85	0.015
Caudal anterior cingulate	Right	16	0	24	134	4.37	0.034
**B. Effect of group**							
**Seed: left STS**							
STG	Right	44	−12	−10	167	5.24	0.012
STG	Left	−46	−34	6	140	4.27	0.028
Postcentral/precentral	Right	14	−34	66	433	4.63	<0.001
Posterior cingulate	Right	10	−42	26	542	4.48	<0.001
**C. Interaction time × congruency in typical readers**							
**Seed: left OT**							
SPL	Right	26	−52	60	241	4.67	0.001
MTG	Left	−58	−16	−12	174	5.54	0.007
**D. Interaction time × congruency in poor readers**							
**Seed: left OT**							
IFG op./ STG	Left	−56	2	2	613	6.90	<0.001
**E. Interaction group × congruency in first grade (T1)**							
**Seed: left OT**							
IFG op./ STG	Left	−56	8	−6	265	5.10	0.001
Precuneus/SPL	Right	10	−64	60	273	4.95	0.001
**Seed: left STS**							
Precuneus/mid cingulum	Left	−4	−40	44	414	4.61	<0.001
Precentral/postcentral	Right	10	−28	66	399	5.86	<0.001
**F. Interaction group × congruency in second grade (T2)**							
**Seed: left OT**							
rolandic operculum/STG/insula	Left	−38	2	0	170	4.70	0.005
Insula/rolandic operculum	Right	38	8	6	130	4.48	0.022
**Seed: left STS**							
STG/IFG op.	Left	−46	20	−16	421	5.82	<0.001

*Cluster extent family-wise error (FWE)-corrected P_FWEc_ < 0.05 on a voxel-level CDT P_uncorr_ < 0.001. T-values and MNI coordinates are given for the peak voxel of each cluster.*

Additional group-wise analyses revealed a significant interaction of congruency by time in typical readers for the coupling between the left OT seed with the right superior parietal lobule (SPL) and the left MTG (Figure 4B and Table 3C). Post hoc t-tests showed that the coupling for AVc pairs increased, while the coupling for AVi pairs decreased from T1 to T2. We also found a significant interaction of congruency by time in poor readers for the coupling between the left OT seed with a cluster around the rolandic operculum encompassing parts of the IFG op., precentral gyrus and STG (including Heschl’s gyrus) (Figure 4B and Table 3D). Post hoc *t*-tests showed that the coupling for AVc pairs decreased in this region, while the coupling for AVi pairs increased over time. No developmental changes within groups were detected in the seed-based analyses using the STS seed.

#### Group Comparisons of FC During Audiovisual Integration in First and Second Grade

**FIGURE 5:**
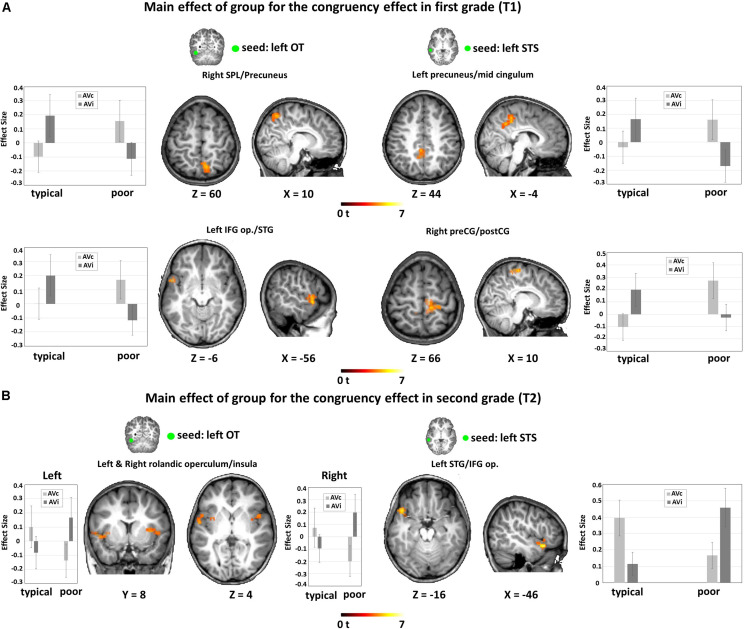
Fc-fMRI analysis: group differences within each time point. **(A)** Main effect of group for the congruency effect in first grade (T1). Results showed increased FC from the seed regions to frontotemporal and parietal regions for AVc pairs in poor readers and for AVi pairs in typical readers. **(B)** Main effect of group for the congruency effect in second grade (T2). Results showed increased FC from the seed regions to temporal regions for AVc pairs in typical readers and for AVi pairs in poor readers. Significant results are reported at a voxel-level uncorrected *P* < 0.001 and a cluster extent family-wise error (FWE)-corrected *P*_*FWEc*_ < 0.05. FC, functional connectivity; OT, occipito-temporal; STS, superior temporal sulcus; SPL, superior parietal lobule; IFG op., inferior frontal gyrus pars opercularis; STG, superior temporal gyrus; preCG, precentral gyrus; postCG, postcentral gyrus.

In first grade, we found a significant interaction effect between congruency and group. This interaction effect was driven by increased functional connectivity for the congruency effect (AVc vs. AVi) in poor readers and an increased functional connectivity for the incongruency effect (AVi vs. AVc) in typical readers between 1) the left OT seed and a cluster in the left temporal pole, extending into the planum temporale and the IFG op., and the left precuneus and 2) the left STS seed and the left precuneus and the right postcentral gyrus (Figure 5A and Table 3E).

We also found a significant interaction effect between congruency and group in second grade. This interaction effect was driven by increased functional connectivity for the congruency effect (AVc vs. AVi) in typical readers and an increased functional connectivity for the incongruency effect (AVi vs. AVc) in poor readers between 1) the left OT seed and bilateral clusters in the insula, extending into the STG and planum temporale in the left hemisphere and 2) the left STS seed and the left STG/IFG (Figure 5B and Table 3F).

## Discussion

In this study, we examined developmental changes related to audiovisual (AV) integration of short written and spoken nonwords from beginning readers in the middle of first grade (T1) to more practiced readers in the middle of second grade (T2). Across the whole sample, no differential activation for congruent and incongruent AV nonwords pairs was found at T1. An incongruency effect, i.e., stronger activations for incongruent (AVi) vs. congruent (AVc) nonwords emerged in bilateral temporal and frontal areas at T2. The statistical comparison over time revealed a significant increase of the incongruency effect (AVi vs. AVc) from T1 to T2 in the right MTG/ITG. When comparing typical with poor reading children at T2, we found a congruency effect (AVc vs. AVi) in the right MFG that was characteristic for poor readers but not typical readers. Additionally, we found that gains in pseudoword reading from T1 to T2 were significantly associated with differential processing of AVc and AVi at T1 and T2, and with developmental changes of these congruency effects in the left STG. Finally, FC between the left OT cortex and the IFG op./STG showed differential developmental patterns for typical and poor readers, with poor readers developing a stronger left OT-IFG op./STG FC for AVi from T1 to T2, an effect that was already present at T1 for typical readers. Left OT-right SPL FC was found to be stronger at T1 for AVi in typical readers than poor readers, with typical readers developing stronger FC for AVc in this region at T2. Further group comparisons within each time point suggested differences in the coupling patterns of the left OT cortex and the left STS to regions of the default mode network (DMN) during AV integration of nonwords. These findings are discussed in detail below.

### Audiovisual Integration in Temporal and Frontal Areas Emerges in Second Graders

The whole brain analysis in the middle of first grade (T1) revealed no differential activation patterns for processing AVc vs. AVi nonwords. This may indicate that AV integration of print and speech was not yet automatized after only a few months of reading instruction at school in the middle of first grade. Interestingly, around one year later in the middle of second grade (T2) we found some early indications of implicit differentiation of matching and nonmatching information, as reflected by stronger activations for AVi than AVc pairs in bilateral inferior temporal gyri (ITG), the left superior and inferior frontal gyri (SFG/IFG) and the right MTG. In particular, the incongruency effect in the right MTG/ITG emerged from first grade to second grade. Activation in the MTG has been linked to phonological processing (Oron et al., 2016) or more directly to grapheme-phoneme decoding (Jobard et al., 2003; Martin et al., 2016). The right ITG has been implicated as a generator of the auditory mismatch negativity and seems to be involved in sound discrimination processes (Waberski et al., 2001; Monzalvo and Dehaene-Lambertz, 2013). Thus, the stronger activation to AVi pairs on the one hand may reflect increasing demands when accessing different phonological information for written and spoken nonwords. On the other hand, this incongruency effect may also relate to the detection of audiovisual mismatch when processing incongruent LS pairs, which becomes increasingly automatized with more reading practice in second grade. Furthermore, the left IFG and SFG are known to be crucial regions in sustaining attention to phonemes (Gelfand and Bookheimer, 2003; Proverbio et al., 2018). Stronger activation to AVi nonwords in these areas may thus be explained by the conflicting grapheme-phoneme information requiring more effort for maintaining phoneme information or directing attention to relevant information.

The incongruency effect for nonwords at T2 bilaterally in the ITG, is of particular interest. The left hemispheric ITG cluster extended to the fusiform gyrus and thus overlapped partly with the ventral temporal cortex which is known to specialize to visually process letters and words (Flowers et al., 2004; Dehaene et al., 2005; Thesen et al., 2012) with reading acquisition (Brem et al., 2010; Dehaene-Lambertz et al., 2018; Pleisch et al., 2019b). Our results suggest that this region develops a sensitivity to implicit AV matching of nonwords in emergent readers at T2, an effect that is not present earlier in beginning readers, after half a year of reading instruction at T1. During reading acquisition, cortical regions that reorganize to process speech and print have been shown to overlap (Dehaene et al., 2015). This overlap suggests that specific regions develop to support both auditory and visual processing of language, and this convergence in form of a print-speech coactivation might serve fast word recognition and therefore fluent reading (Preston et al., 2016).

In addition, an interaction effect of congruency and group was found in the right MFG in T2. While both groups showed deactivation in the MFG for AVc and AVi, the interaction indicated a more pronounced deactivation for AVi compared to AVc in poor readers, the typical readers showed a similar deactivation for both conditions. The MFG has been previously associated with attentional control and as part of a multiple demand system controlling different parts of diverse cognitive demands (Duncan, 2010). Involvement of the MFG in reading may in general indicate recruitment of additional executive resources (Meyler et al., 2008; Arrington et al., 2019) and has been found to be less involved in phoneme identification in readers with dyslexia than in typical readers (Beneventi et al., 2010). In the current study however, we observed negative BOLD effects in the right MFG during audiovisual processing. Negative BOLD signals could reflect reduced neuronal processing as compared with baseline and have been related to the suppression of specific cognitive processes (Shmuel et al., 2002; Amedi et al., 2005). Our finding may thus be a tentative indication that the allocation of attention as directed through rMFG involvement is suppressed specifically during processing incongruent LS pairs in poor reading children.

### Audiovisual Integration in the Temporo-Parietal Cortex Is Related to Reading Outcome

In a previous study, AV integration of single LS pairs was initiated with a short artificial grapheme-phoneme training, but significantly depended on learning performance (Karipidis et al., 2017). Here, we studied how improvements in decoding skills as measured with pseudoword reading were associated with AV integration of short pronounceable nonwords. We found that the AV congruency effect (AVc vs. AVi) changed as a function of pseudoword reading performance within each time point and across time. More specifically we observed a shift from an incongruency effect (negative AVc vs. AVi difference, AVi > AVc) toward a congruency effect (positive AVc vs. AVi difference, AVc > AVi) with higher pseudoword reading performance improvements for both time points in a network of parietal, temporal, occipital, and frontal brain regions.

In an early reading stage, in first grade, a stronger congruency effect bilaterally in the SPL was associated with higher pseudoword reading performance improvements from T1 to T2. Increased functional responses in the left SPL, as part of the phonology mediated pathway for decoding of written input, and the right SPL, as part of the dorsal attention network, have been consistently found for age-matched skilled readers compared with readers with dyslexia (Hoeft et al., 2006; van der Mark et al., 2009; Church et al., 2011). Involvement of the SPL may thus support the successful development of reading fluency and rapid decoding of pseudowords.

Higher pseudoword reading performance improvements from first to second grade were associated with stronger congruency effects bilaterally in the STG in second grade. These effects are in line with studies reporting that higher literacy skills correlate with stronger congruency effects during AV speech perception (Nath et al., 2011) in the superior temporal cortex. The STS/STG is critical for the integration of AV features (Beauchamp et al., 2004), of LS pairs (Holloway et al., 2015; for a review, see Richlan, 2019) and conceptual matching (Hocking and Price, 2008). The positive association of pseudoword reading skills with the congruency effect in the left STG significantly increased from first to second grade, suggesting that the sensitivity of the STG to AV integration depends on the skill of fluently decoding LS combinations.

Overall, our results suggest that the automatic matching of incoming written and spoken nonword input is modulated by children’s improvement in reading performance and starts early, i.e., in the middle of first grade (T1) with emerging recruitment of attentional and phonological systems to congruent AV information in the superior parietal lobe. Later on, with more expertise in middle of second grade (T2), additional regions in the superior temporal, middle frontal, and postcentral cortex show increased sensitivity to congruent AV information, with the strongest developmental alterations of the congruency effect being evident in the left STG.

### Development of FC in Left OT Cortex During AV Integration

Our findings indicate different developmental patterns of FC during AV integration of nonwords in typical and poor readers. Both groups showed significant changes in FC for the OT seed during AV integration (i.e., AVc vs. AVi) from T1 to T2. Of note, the largest cluster that showed a significant interaction effect of time by group for the left OT seed, spanned the IFG op., the STG and parts of the insula. While typical readers showed stronger connectivity of OT-IFG op./STG for AVi in first grade, in poor readers this effect was observed later on in second grade. More specifically, beginning readers with poor reading skills showed a developmental change of the task-based functional coupling from a congruency effect at T1 (AVc > AVi) toward an incongruency effect at T2 (AVi > AVc). The FC patterns between two core regions of the neural circuit of reading thus show pronounced developmental changes for AV processing that depend on reading skills. This finding of altered FC in poor readers converges with previous reports of disrupted FC between the left IFG and the left OT cortex in adolescents and children with dyslexia during task-based (Olulade et al., 2015; Morken et al., 2017) and resting state fMRI (Schurz et al., 2015).

Children with typical reading skills in our study showed a developmental increase in coupling between the left OT cortex and the right SPL from T1 to T2 for AVc pairs, while the coupling between these regions for AVi pairs decreased over time. As noted before, the SPL has been implicated in visual attention span (Peyrin et al., 2011, 2012) and serial decoding (Richlan, 2014) in particular when processing nonword letter strings rather than single letters, whereby the right SPL seems more sensitive to attentional demands than the left counterpart (Lobier et al., 2012). Likewise, the more demanding attentional shifting for processing smaller letter chunks in nonwords as compared to words (Vogel et al., 2012) was reflected by enhanced activation of the dorsal attention network for nonwords (Church et al., 2011). Our data are consistent with the findings of significant task-based FC between ventral OT cortex and SPL in typical readers as compared to children with dyslexia (van der Mark et al., 2009). The data also converge with previous findings suggesting that early OT-parietal FC during a rhyming task is associated with later increases in reading skills (Younger et al., 2017) and with the increasing correlation between resting state FC between the ventral OT cortex and SPL as a function of age and reading skills (Vogel et al., 2012). More resources may be allocated to AVc items, which then support the serial decoding of letter strings and thus integration of AV information. Taken together, our finding, along with others, suggest the increasing contribution of the dorsal attention network in processing letter strings with development and reading practice through the coupling with the left occipito-temporal cortex.

### Altered FC During AV Integration in Poor vs. Typically Reading First Grade Children

In first grade, poor readers showed stronger connectivity for the congruency effect (AVc vs. AVi) while typical readers showed stronger connectivity for the incongruency effect (AVi vs. AVc) between the seed regions (left OT and STS) and areas of the default mode network (DMN), in particular the precuneus (and for STS FC also to parts of PCC). Specifically, both seed regions showed significantly diminished coupling/increased decoupling for the AVi items in poor reading children to the precuneus, explaining the increased coupling for the congruency effect in this group. In contrast, AVc items showed increased decoupling for the typical readers with this DMN region. Our results suggest that a functional segregation (decoupling) of our seed regions to DMN regions when processing AVc and a coupling when processing AVi information characterizes the connectivity pattern of emergent readers with typical but seems reversed in children with poor reading development.

These findings support the assumption that automatized reading not only necessitates strong functional coupling between language regions (Koyama et al., 2011; Schurz et al., 2015) but also functional segregation, i.e., negative coupling, of language processing regions with the DMN, such as the precuneus and PCC (Koyama et al., 2011). Even though the study by Koyama et al. (2011) indicates functional segregation of OT and DMN (precuneus) in the adult brain but not in developing children, the study of Schurz et al. (2015) showed clear associations of increased functional coupling of language areas to the DMN (e.g., PCC/precuneus) in children with dyslexia in task-based FC. Aberrant patterns of connectivity have thus equally been described for resting state and task-based data (e.g., phonological lexical task in Schurz et al., 2015, rhyming tasks in Cao et al., 2008; Finn et al., 2014). The high correspondence between task-based and resting state fMRI data in the functional connectivity with DMN (Mennes et al., 2010) emphasize the necessity of functional segregation of language processing regions with the DMN (PCC/precuneus) in automatized reading.

In addition to the coupling with the DMN, we also found increased connectivity of the seed regions with reading related areas. FC from the left OT to the IFG op./STG showed increased coupling for AVi in typical readers and for AVc in poor readers, consistent with the developmental effect described above. FC from the left STS to the precentral gyrus, extending to the postcentral gyrus, followed the same coupling pattern, with poor readers showing increased decoupling for AVi and increased coupling for AVc pairs. The left (Bach et al., 2010) and right (Paulesu et al., 2014) precentral gyrus showed consistent overactivation in children and adults with dyslexia as reported in meta-analyses (e.g., Richlan et al., 2009, 2011). This effect seemed to be especially pronounced in relatively shallow orthographies such as German (Martin et al., 2016). The overactivation commonly seen in poor readers in these regions is usually assumed to reflect compensational reliance on subvocal articulatory rehearsal (Price, 2012; Richlan, 2014; Hancock et al., 2017). Our experimental task did not require explicit processing of the short nonwords, with the participants main task being the detection of targets. Nevertheless, what can be considered as simple decoding for an experienced adult reader, is in fact a very complex skill and represents an effortful task especially for poor reading children in first and second grade. Therefore, the increased functional coupling with bilateral precentral/postcentral gyri for AVc items may represent compensatory reliance on articulation and rehearsal that is associated with poor reading performance in emergent readers.

### Altered FC During AV Integration in Poor vs. Typically Reading Second Grade Children

In second grade, poor reading children showed increased FC for AVi pairs between the left OT seed and bilateral insular cortex as well as left STS and a larger region spanning the frontal opercular cortex and the left temporal pole compared with typical readers. These results suggest an increased link to phonological and lexical-semantic areas when poor reading children process AVc information and at the same time a decreased link to these areas when typically reading children process nonmatching AVi information. A stronger coupling between left fusiform gyrus and the left insula, and stronger insula activation during a visual phonological lexical decision task was previously reported for controls compared to children with developmental dyslexia (van der Mark et al., 2009, 2011). The insula contributes to several key language processes, including phonological processing and visual-auditory integration (Bamiou et al., 2003) and the congruency and time dependent change in FC of left OT cortex to bilateral insulae may thus indicate differential contributions to multisensory integration and phonological verbal short-term memory processes in our study.

Left frontal opercular sites are thought to specifically support the processing of sublexical or phonological segmentation (Burton et al., 2000) especially at the level of phonemes. This is consistent with the finding of more activation in the left frontal opercular sites when subjects read pronounceable nonwords as compared with words (e.g., high frequency consistent and inconsistent words, Fiez et al., 2006). It has been suggested that increased opercular activation might be associated with higher processing load when reading nonwords and irregular words, because whole-word processing is not possible and instead phonological assembly processes are used (Twomey et al., 2015). Hence, the association of increased connectivity with the left frontal opercular regions in poor readers may indicate more involvement of phonological processing effort for AVi nonword information.

### Limitations

Our longitudinal approach of exploring AV processing at the emergent reading stage, middle of the first grade, and one year later in the middle of second grade provides novel insights on the development of integrating print and speech during the initial learning phase in school children. However, several limitations of our study should be addressed. Firstly, we examined a sample of beginning readers all having a moderate or high familial risk for developmental dyslexia according to the ARHQ or their language development. Although such samples are of particular importance to better understand divergent developments of functional activation and connectivity patterns related to reading problems, the results of such a risk-sample cannot easily be generalized to the typically developing population. In addition, there is ample evidence for behavioral and brain differences in at-risk children even before the start of formal education including microstructural alterations, altered functional activation or event-related potentials (Maurer et al., 2003, 2007, 2009; Guttorm et al., 2005; Specht et al., 2009; Yamada et al., 2011; Raschle et al., 2012; Bach et al., 2013; Brem et al., 2013; Dȩbska et al., 2016; Karipidis et al., 2017, 2018; Centanni et al., 2018; Thiede et al., 2019; De Vos et al., 2020). Further studies including non-risk samples may clarify whether some developmental alterations in AV integration are specific to children at risk for developmental dyslexia.

Secondly, the small sample size especially for the group analyses restricted the statistical power of the reported results. The limited number of poor readers did not allow to investigate potential heterogeneity in the cognitive deficits that could be associated with poor reading within this group. Replication studies are thus urgently needed to verify the effects seen in our small groups and address further research questions. Finally, similar with several other pediatric studies (Wimmer et al., 2010; Raschle et al., 2012; Dȩbska et al., 2016; Wang et al., 2018), the statistical threshold defined for reporting significant results of whole brain BOLD analyses is liberal. Reporting the results of uncorrected whole brain analyses includes the risk of interpreting false positive activations. Applying the threshold used here on the other hand reduces the risk of missing potentially meaningful functional alterations (Wimmer et al., 2010). Nevertheless, we believe that the uncorrected results of our whole brain analyses provide interesting insights into potential developmental alterations between groups and with development but should be interpreted with care and need replication in independent studies.

## Conclusion

In conclusion, functional connectivity and BOLD activation analyses both point to developmental changes related to audiovisual integration in beginning readers at risk for developmental dyslexia and with varying reading skills. The developmental changes were reflected in reading skill dependent alterations in the brain responses to congruent and incongruent nonword pairs in temporo-parietal regions, which are known to be critically involved in audiovisual processing. Functional coupling between the left occipito-temporal seed region and bilateral IFG op./STG showed group dependent developmental differences, with poor readers showing a significant increase of connectivity for AVi compared to AVc over time. Moreover, in typical readers functional coupling between the left occipito-temporal seed region and the dorsal parietal attention network increased over time for AVc and decreased for AVi nonword pairs. This sheds new light on the differences in the development and organization of functional circuits during audiovisual matching in emergent readers at risk for dyslexia.

## Data Availability Statement

The datasets generated for this study are available on request to the corresponding author.

## Ethics Statement

This study involving human participants was reviewed and approved by Kantonale Ethikkommission Zürich and neighbouring Cantons in Switzerland. Written informed consent to participate in this study was provided by the participants’ legal guardian/next of kin.

## Author Contributions

SB, IK, GP, and FW: conceptualization. IK and GP: data collection. FW, IK, and SB: formal analysis, writing – original draft. FW, IK, GP, and SB: investigation. FW, IK, GP, GF-G, and SB: methodology. SB: supervision. SB, IK, GF-G, and GP: writing – revision and editing. All authors contributed to the article and approved the submitted version.

## Conflict of Interest

The authors declare that the research was conducted in the absence of any commercial or financial relationships that could be construed as a potential conflict of interest.

## Footnotes

1 www.neurobs.com

## Supplementary Material

TABLE S1 Head motion during the fMRI task: mean scan-to-scan (framewise) displacement for the six realignment parameters.
