# Supplementary material for: Development of Print-Speech Integration in the Brain of Beginning Readers With Varying Reading Skills
Source: Front Hum Neurosci. 2020 Aug 14;14:289. doi: 10.3389/fnhum.2020.00289 (PMC7457077; doi:10.3389/fnhum.2020.00289)
Supplement: TABLE S1 — Head motion during the fMRI task: mean scan-to-scan (framewise) displacement for the six realignment parameters. [file Table_1.docx]

**Supplementary Table S1.** Head motion during the fMRI task: mean scan-to-scan (framewise) displacement for the six realignment parameters

|  |  | T1 | | T2 | |
| --- | --- | --- | --- | --- | --- |
|  |  | M ± SD  (Range) | | M ± SD  (Range) | |
|  |  | typical | poor | typical | poor |
| Translation (mm) | x | 0.031972 ± 0.033749  (0.009332 – 0.153166) | 0.020673 ± 0.008125  (0.010621 – 0.034698) | 0.025864 ± 0.017425  (0.006584 – 0.062158) | 0.022502 ± 0.01371  (0.011833 – 0.068624) |
|  | y | 0.050542 ± 0.020753  (0.022323 – 0.091017) | 0.043127 ± 0.019111  (0.022438 – 0.079626) | 0.047264 ± 0.018929  (0.025422 – 0.089912) | 0.04164 ± 0.016683  (0.017208 – 0.06724) |
|  | z | 0.080123 ± 0.040098  (0.025678 – 0.181216) | 0.071252 ± 0.028089  (0.029124 – 0.124249) | 0.091931 ± 0.050954  (0.024286 – 0.194538) | 0.075669 ± 0.032001  (0.040122 – 0.135413) |
| Rotation (degree) | pitch | 0.001197 ± 0.000527  (0.00608 – 0.002692) | 0.001134 ± 0.00036  (0.000624 – 0.001716) | 0.001249 ± 0.000453  (0.000491 – 0.002056) | 0.001127 ± 0.000388  (0.000522 – 0.001718) |
|  | roll | 0.000647 ± 0.000423  (0.000227 – 0.002001) | 0.000468 ± 0.000199  (0.00231 – 0.000913) | 0.000564 ± 0.000387  (0.00023 – 0.001497) | 0.000555 ± 0.00048  (0.000276 – 0.002256) |
|  | yaw | 0.000444 ± 0.000509  (0.000193 – 0.002328) | 0.000288 ± 0.000113  (0.000184 – 0.000401) | 0.000328 ± 0.000221  (0.000116 – 0.000753) | 0.000285 ± 0.00015  (0.00014 – 0.000757) |

Note: T1: first grade; T2: second grade; M: mean; SD: standard deviation.
